# Occurrence of Nontuberculous Mycobacterial Pulmonary Infection in an Endemic Area of Tuberculosis

**DOI:** 10.1371/journal.pntd.0002340

**Published:** 2013-07-18

**Authors:** Ana Roberta Fusco da Costa, Joseph O. Falkinham, Maria Luiza Lopes, Adriana Rodrigues Barretto, João Soares Felicio, Lúcia Helena Messias Sales, Jeann Ricardo da Costa Bahia, Emilyn Costa Conceição, Karla Valéria Batista Lima

**Affiliations:** 1 Bacteriology and Mycology Section, Evandro Chagas Institute, Ananindeua, Para, Brazil; 2 Department of Biological Sciences, Virginia Polytechnic Institute and State University, Blacksburg, Virginia, United States of America; 3 João de Barros Barreto University Hospital, Federal University of Para, Belem, Para, Brazil; 4 Department of Integrative Medicine, Federal University of Para, Belem, Para, Brazil; University of Tennessee, United States of America

## Abstract

The majority of investigations of the epidemiology of nontuberculous mycobacteria (NTM) have focused on highly developed nations with a low prevalence of tuberculosis. In contrast, the Para state of north Brazil represents an area of high tuberculosis prevalence and increasing NTM incidence. Toward the goal of understanding the dynamics of infection by all *Mycobacterium* species, we report patient characteristics and the identification of NTM strains isolated from sputum samples from patients that were residents of Para, a state in the Amazon region, Northern of Brazil, over the period January 2010 through December 2011 (2 years). The 29 NTM patients comprised 13.5% of positive mycobacterial cultures over the 2-year period. A major risk factor for NTM pulmonary disease was previous tuberculosis (76%). Further, the average age of NTM patients (52 years) was significantly higher than that of tuberculosis patients (39 years) and more were female (72.4% vs. 37.4%). Unlike other Brazilian states, NTM pulmonary patients in Para were infected with a different spectrum of mycobacteria; primarily the rapidly growing *Mycobacterium massiliense* and *Mycobacterium simiae* complex.

## Author Summary

Nontuberculous mycobacteria (NTM) are environmental organisms that are naturally found in soil, water, dust and other sites. Several case reports and studies on the prevalence of pulmonary NTM disease have been published, nevertheless, the impact and the exact magnitude of NTM infections in countries where tuberculosis (TB) is endemic are not known. Here, we report the identification of NTM strains isolated from pulmonary samples from patients with a presumptive diagnosis of pulmonary TB and residents of the State of Para, in the Amazon region, Northern of Brazil. This study documents the occurrence and diversity of species of NTM that cause pulmonary disease in a region representative of those in the world with high infection rates by *Mycobacterium tuberculosis*.

## Introduction

Nontuberculous mycobacteria (NTM) are environmental opportunistic pathogens that are natural inhabitants of soil [1] and drinking water [2], [3]. Humans and their agronomic animals are literally surrounded by nontuberculous mycobacteria [4]. Risk factors for NTM pulmonary disease include: prior tuberculosis, chronic obstructive pulmonary disease (COPD), lung damage due to occupational exposures to dusts (e.g., mining), cystic fibrosis or heterozygosity for a cystic fibrosis mutation, α-1-antitrypsin deficiency [5]. Fisherman and others exposed to fish are at risk for skin infections caused by *Mycobacterium marinum* infection [5] and children from 18 months to 5 years of age are at risk for cervical lymphadenitis caused more typically by *M. avium* [6]. Immunodeficiency, due to HIV-infection or immunosuppression due to cancer or chemotherapy are risk factors for *Mycobacterium avium* bacteremia [2].

Several case reports and studies on the prevalence of pulmonary disease caused by NTM in North America, Europe and Japan have been published during recent years [7], [8], [9], [10], [11]. Nevertheless, the impact and the exact magnitude of NTM infections in countries where tuberculosis is endemic are not known. Here, we report the identification of NTM strains isolated from pulmonary samples from patients with a presumptive diagnosis of pulmonary TB and residents of the State of Para, in the Amazon region, Northern of Brazil. This study documents the occurrence and diversity of species of NTM that cause pulmonary disease in a region representative of those in the world with high infection rates by *M. tuberculosis*.

## Materials and Methods

### Patients and Clinical Samples

Patients from routine laboratory presenting symptoms suggestive of mycobacterial disease (e.g., chronic cough) and/or who were noted to have radiological alterations at medical examination, and from NTM were isolated at least once between January 2010 and December 2011 at the Evandro Chagas Institute, were included in this study. All the NTM isolates described in this study were obtained from pulmonary samples (sputum, bronchoalveolar washes, and gastric washes samples) of 38 individuals residents of the State of Para, North Brazil. Patient records were reviewed to assess the clinical data. Diagnostic criteria for NTM disease published by the American Thoracic Society (ATS) were applied to determine the clinical relevance of NTM isolation (Table 1) [12].

**Table 1:** American Thoracic Society diagnostic criteria on nontuberculous mycobacterial pulmonary disease.

**Clinical and radiographic**
Pulmonary symptoms, nodular or cavitary opacities on chest radiograph, or an high resolution computed tomography (HRCT) scan that shows multifocal bronchiectasis with multiple small nodules and; Appropriate exclusion of other diagnoses
**Microbiologic**
Positive culture results from at least two separate expectorated sputum samples. (If the results from the initial sputum samples are non-diagnosed, consider repeat sputum acid-fast bacilli (AFB) smears and cultures) or; Positive culture results from at least one bronchial wash or lavage or;
**Histopathologic**
Transbronchial or other lung biopsy with mycobacterial histopathologic features (granulomatous inflammation or AFB) and positive culture for NTM or biopsy showing mycobacterial histopathologic features (granulomatous inflammation or AFB) and one or more sputum or bronchial washings that are culture positive for NTM.

Note: Table adapted from Griffith et al. [12].

The clinical samples were initially decontaminated, using the N-acetyl-L-cysteine-sodium hydroxide procedure [13]. The samples were subsequently inoculated onto Löwenstein–Jensen medium (Difco, Sparks, USA) and incubated at 35°–37°C in the absence of light for at least six weeks or until colonies appeared. Isolates of the *M. tuberculosis* complex were distinguished from NTM by the unique breadcrumb or cauliflower colony morphology of *M. tuberculosis*, and the production of cord factor and susceptibility to 0.5 mg/mL of para-nitrobenzoic acid by *M. tuberculosis* [14].

### Ethics Statement

All subjects provided written consent by signing the free and informed consent form, and all patients data analyzed were anonymized. This study was approved by the ethics committee of the Evandro Chagas Institute (protocol n° 017/10, CAAE: 0017.0.072.000-10).

### Molecular Identification

All NTM isolates of this study were identified by sequencing a portion of the 16S rRNA [15] and *hsp65* [16] genes.

### Statistical Analysis

The descriptive analysis were expressed as mean ± standard deviation or percentage, while analytical statistics was conducted using either non-parametric Chi-squared test or G-test, using the software BioEstat version 5.01 [17]. Statistical significance was defined as *p*<0.05.

## Results

### Patient Characteristics

From January 2010 to December 2011, a total of 69 NTM isolates were recovered from pulmonary specimens from 38 patients with respiratory symptoms that included chronic cough and alterations on chest X-ray. The patients and their characteristics are listed in Table 1. The 38 patients represented 13.5% of culture-positive mycobacterial cultures obtained in our laboratory over that period of time. Of the 38 patients, 29 met the American Thoracic Society diagnostic criteria for NTM infection [12]. All patients were initially diagnosed as having pulmonary tuberculosis (*M. tuberculosis*) based on sputum smear microscopy for acid-fast bacilli (AFB) and had suffered a treatment failure.

**Figure 1:**
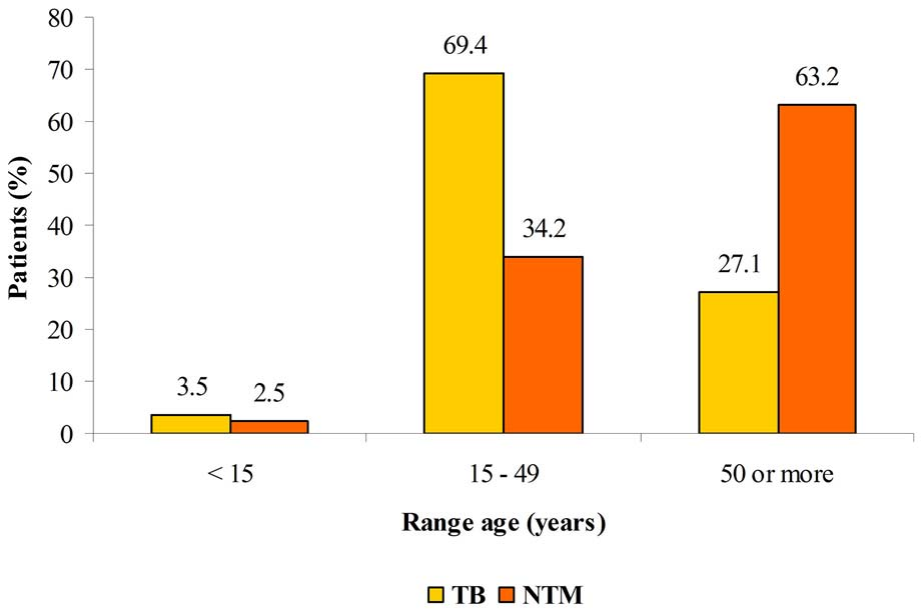
Proportion of tuberculosis and nontuberculous mycobacterial lung disease by age group - State of Para, Brazil, 2010–2011.

A summary of the characteristics of the 29 NTM patients meeting the criteria for NTM disease is provided in Table 2. Among the patients with NTM disease the mean age was 52.3 years (±17.8 SD), and the mean time from onset of symptoms to NTM diagnosis was of 7.8 months (±13.5 SD). Twenty of the 29 patients (68.9%) were above 50 years old, whereas 69.4% of tuberculosis cases (X^2^ = 26.7; *p*<0.0001) reported in the state of Para are under 50 years old (Figure 1).

**Table 2:** Individuals who met the ATS microbiological criteria for nontuberculous mycobacterial pulmonary disease from Para State, Brazil.

N°	Race/color category	Gender	Age	Occupation	Water source	Area	AFB smear	N° positive cultures	Clinical specime	HIV	Other associated conditions	Species
1	Pardo	F	52	Pensioner	Piped system	Urban	−	2	Sputum	−	Prior pulmonary TB	*M. intracellulare*
2	Pardo	F	25	Housewife	Piped system	Urban	+	3	Sputum	+	Prior pulmonary TB	*M. avium*
3	Pardo	F	54	Housewife	Well	Urban	+	3	Sputum	−	Prior pulmonary TB; exposure to biomass smoke; bronchiectasis	*M. massiliense*
4	White	F	55	Housewife	Piped system	Urban	−	3	Sputum	−	Prior extrapulmonary TB; bronchiectasis; cavitary lung lesions; long-term corticosteroids use	*M. massiliense*
5	Pardo	F	71	Housewife	Piped system	Urban	+	2	Sputum	−	Prior extrapulmonary TB; bronchiectasis; cavitary lung lesions	*M. massiliense*
6	Pardo	F	32	Housewife	Well	Rural	+	2	Sputum	+	Ethilism; smoking	*M. avium*
7	Pardo	F	34	Housewife	Piped system	Urban	−	3	Sputum	−	Down syndrome	*MSC* (unspeciated)
8	Pardo	F	42	Craftswoman	Well	Rural	+	3	Sputum	−	Bronchiectasis; cavitary lung lesions	*M. abscessus*
9	White	F	64	Pensioner	Piped system	Urban	+	3	Sputum	−	Prior pulmonary TB	*M. massiliense*
10	Pardo	M	62	Farmer	Well	Rural	−	2	Sputum	−	Bronchiectasis; cavitary lung lesions; COPD; smoking	*MSC* (unspeciated)
11	Pardo	F	19	Student	Piped system	Urban	−	2	Sputum	−	Prior pulmonary TB; bronchiectasis	*M. intracellulare*
12	Pardo	F	53	Housewife	Well	Urban	+	2	Sputum	−	Prior pulmonary TB	*M. massiliense*
13	White	F	69	Pensioner	Piped system	Urban	+	2	Sputum	−	Prior pulmonary TB; cavitary lung lesions	*M. abscessus*
14	Pardo	F	41	Uninformed	Piped system	Urban	+	3	Sputum	−	Prior pulmonary TB; cavitary lung lesions	*M. massiliense*
15	Pardo	M	9	Student	Piped system	Urban	−	1	GL	+	Prior pulmonary TB; cavitary lung lesions	*M. avium*
16	Pardo	F	58	Housewife	Piped system	Urban	+	3	Sputum	−	Prior pulmonary TB; cavitary lung lesions	*M. intracellulare*
17	Pardo	M	61	Broker	Piped system	Urban	+	1	BAL	−	Prior pulmonary TB	*M. massiliense*
18	Pardo	F	56	Housewife	Piped system	Urban	+	3	Sputum	−	Prior pulmonary TB; cavitary lung lesions	*M. massiliense*
19	Pardo	F	84	Farmer	Well	Rural/Island	+	2	Sputum	−	Prior pulmonary TB	*M. massiliense*
20	Pardo	F	63	Housewife	Well	Urban	+	2	Sputum	−	Chronic bronchitis	*M. massiliense*
21	Pardo	M	53	Uninformed	Piped system	Urban	−	1	BAL	−	Prior pulmonary TB; hematologic neoplasia; bronchiectasis	*M. massiliense*
22	Pardo	F	60	Seamster	Piped system	Urban	+	2	Sputum	−	Rheumatoid arthritis; long-term corticosteroids use; bronchiectasis; smoking	*M. bolletii*
23	Pardo	M	64	Carpenter	Well	Rural	+	2	Sputum	−	Prior pulmonary TB; bronchiectasis; smoking	*M. celatum*
24	Pardo	M	33	Civil servant	Well	Rural/Island	+	3	Sputum	+	Prior pulmonary TB; smoking	MSC (unspeciated)
25	Pardo	F	33	Housewife	Piped system	Urban	−	2	Sputum	−	Prior pulmonary TB;	*M. moriokaense*
26	Pardo	F	77	Uninformed	Piped system	Urban	+	1	BAL	−	Prior pulmonary TB; bronchiectasis	*M. massiliense*
27	Pardo	F	72	Pensioner	Piped system	Urban	−	2	Sputum	−	Hypothyrodism	*M. fortuitum*
28	Pardo	M	62	Farmer	Well	Rural	+	2	Sputum	−	Prior pulmonary TB; diabetes; asthma; bronchiectasis; cavitary lung lesions	*M. massiliense*
29	Pardo	M	60	Farmer	Well	Rural	+	1	Sputum		Prior pulmonary TB; bronchiectasis; fungus ball	*M. kansasii*

Note: F (female); M (male); AFB (acid fast bacilli); − (negative); + (positive); BAL (bronchoalveolar lavage); GL (gastric lavage); TB (tuberculosis); COPD (chronic obstructive pulmonary disease); MSC (*Mycobacterium simiae* complex).

**Figure 2:**
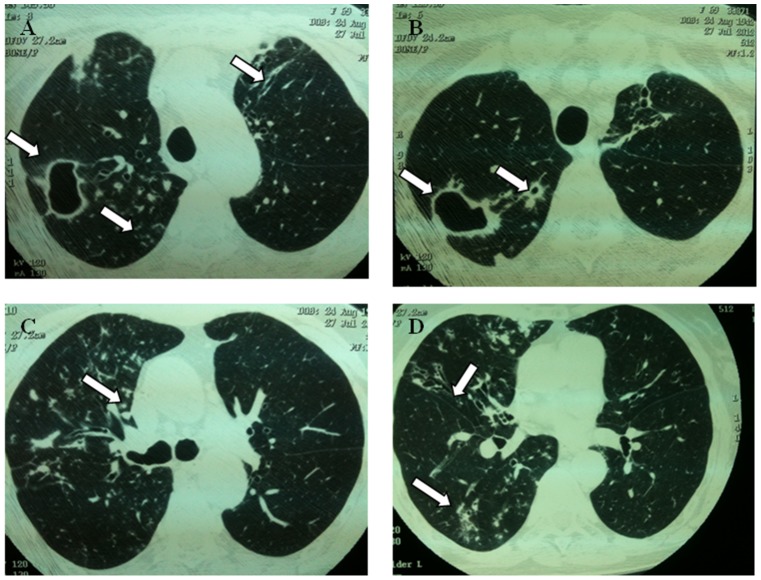
Representative image showing lung damage in a patient with nontuberculous mycobacterial diseases. A 69-years-old woman with *Mycobacterium abscessus* pulmonary disease. (A and B) High resolution computed tomography (HRCT) of the chest obtained at level of upper lobes showing multiples cavities in the right upper lobe and centrilobular nodules. It is also possible to observe bronchiectasis in left upper lobe (arrow). (C) Tree-in-bud pattern. (D) Presence of bronchiectasis in middle lobe. Also note centrilobular nodules at right lower lobe.

Among the most frequent co-morbidities found were prior tuberculosis (22/29, 75.8%) and bronchiectasis (13/29, 44.8%) (Table 2). The results of chest X-ray (CXR) and high resolution computerized tomography (CT) examination are shown in Table 3 and Figure 2. A number of 17 CXR and 12 CT findings of these 29 NTM-patients were reviewed. Atelectasis (12/17, 70.5%) and cavities (7/17, 41.2%) were the most frequent findings in CXR, while bronchiectasis (12/12, 100%), centrilobular nodules/tree-in-bud (8/12, 66.6%) and cavities (6/12, 50%) were more frequently observed in the CT. Pleural thickening was detected in 8 (47.0%) patients. Clinical manifestations of advanced lung disease, such as dyspnea and haemoptysis, occurred in 15 (88.2%) patients.

**Table 3:** Clinical and images findings for some nontuberculous mycobacterial pulmonary infected patients from Para State, Brazil.

N°	General symptoms	Chest X-ray	High resolution computerized tomography of the chest
3	Fever, weight loss, cough, sputum, dyspnea, hemoptysis, chest pain, wheezing	Interstitial opacities (RUL); atelectasis (RUL)	Interstitial opacities (RUL, LUL and LLL); bronchiectasis (RUL, LUL and LLL); bilateral pleural thickening; mosaic attenuation pattern
6	Cough, sputum, dyspnea, chest pain	Acinar opacities (RML); nodules (RUL, RML and LUL)	No data
8	Fever, weight loss, cough, sputum, dyspnea, hemoptysis, clubbing	Interstitial opacities (RUL, RML, RLL, LUL and LLL); atelectasis (RUL); cavity (LUL)	No data
9	Weight loss, cough, sputum	Atelectasis (RUL); pleural thickening	No data
10	Weight loss, cough	Interstitial opacities (LUL); atelectasis (LUL); pleural thickening; bullae	Interstitial opacities (RUL and LLL); cavity (LUL); bronchiectasis (LUL); centrilobular nodules; pleural thickening; emphysema; cysts (RUL, RML and LUL)
11	Weight loss, cough, sputum, dyspnea, hemoptysis, chest pain, wheezing	Interstitial opacities (RUL and LUL); cavity (LUL); bronchiectasis (RUL and LUL)	No data
13	Fever, cough, sputum, dyspnea, hemoptysis, chest pain	Interstitial opacities (RUL and LUL); cavities (RUL)	Acinar opacities (RUL, RML and RLL); cavities (RUL); bronchiectasis (RUL and RML); centrilobular nodules; tree-in-bud
14	Fever, weight loss, cough, sputum, dyspnea, hemoptysis, chest pain	Interstitial opacities (RUL and LUL); atelectasis (RUL); cavity (RUL); pleural thickening	Interstitial opacities (RUL, RLL and LUL); bronchiectasis (RUL and LUL); pleural thickening
16	Fever, weight loss, cough, sputum, dyspnea, wheezing	Interstitial opacities (RUL, RML, RLL and LLL); atelectasis (RML); cavity (RUL); pleural thickening	Interstitial and acinar opacities (RUL, RML, RLL and LUL); cavities (RUL and LUL); bronchiectasis (RUL, RML, RLL and LUL); centrilobular nodules; tree-in-bud; pleural thickening
18	Fever, weight loss, cough, sputum, dyspnea, hemoptysis, chest pain	Interstitial opacities (RUL, RML and LUL); atelectasis (LUL); cavities (RML and LUL); pleural thickening	Interstitial opacities (RUL, RML and LUL); cavity (LUL); bronchiectasis (RUL, RML, LUL and LLL); centrilobular nodules; tree-in-bud; pleural thickening; atelectasis (LUL)
19	Cough, hemoptysis, chest pain	Acinar and interstitial opacities (RUL and LUL); atelectasis (RUL and LUL); pleural thickening	Acinar opacities (RUL and LUL); cavities (RUL); bronchiectasis (RUL and LUL); centrilobular nodules; nodules; pleural thickening
20	Fever, weight loss, cough, sputum, dyspnea, hemoptysis, chest pain, wheezing, clubbing	Interstitial opacities (RUL and LUL); atelectasis (RUL and LUL); pleural thickening; bronchiectasis	Interstitial opacities (RUL, RML, RLL, LUL and LLL); bronchiectasis (RUL, RML and LUL); pleural thickening; cysts
22	Fever, cough, sputum, dyspnea, hemoptysis	Interstitial opacities (RUL, RML, RLL, LUL and LLL); atelectasis (RUL); pleural thickening; bronchiectasis (RUL, RML and LUL)	Interstitial and acinar opacities (RUL, RML, RLL and LUL); bronchiectasis (RUL, RML, RLL, LUL and LLL); centrilobular nodules; tree-in-bud; halo sign
23	Weight loss, cough, sputum, dyspnea, hemoptysis, chest pain, clubbing	Interstitial opacities (RUL, RML, RLL, LUL and LLL); atelectasis (LUL); pleural thickening; bronchiectasis	Interstitial opacities (RUL, RML, RLL, LUL and LLL); bronchiectasis (RUL, RML, RLL, LUL and LLL); pleural thickening; emphysema; cysts
25	Fever, weight loss, cough, sputum, dyspnea, hemoptysis, chest pain	Acinar opacities (LLL)	No data
28	Weight loss, cough, sputum, dyspnea, hemoptysis, wheezing	Interstitial opacities (RUL, RML, RLL, LUL and LLL); cavity (RUL)	Interstitial opacities (RUL, RML, RLL, LUL and LLL); cavities (RLL and LUL); bronchiectasis (RUL, RML, RLL, LUL and LLL); nodules; centrilobular nodules; tree-in-bud
29	Weight loss, cough, sputum, dyspnea, hemoptysis, chest pain, clubbing	Interstitial opacities (RUL, RML, RLL, LUL and LLL); atelectasis (RUL and RML); pleural thickening	Interstitial and acinar opacities (RUL); bronchiectasis (RUL, RML, RLL and LUL); nodules (RLL); centrilobular nodules; tree-in-bud; pleural thickening; cysts; fungus ball

Note: RUL (right upper lobe); LUL (left upper lobe); RML (right middle lobe); RLL (right lower lobe); LLL (left lower lobe).

A total 26 out of the 29 NTM-infected patients (89.6%) were classified as pardo, a Brazilian term for people of mixed white and indigenous heritage, who constitute the majority of the Para state population, with a total of 5,270,307 (69.5%) of the population in the 2010 Brazil Census [18]. The frequency of pardo individuals with NTM was significantly different from the percentage of pardo in the state of Para (X^2^ = 5.5; *p* = 0.0312) (Table 1). Among the 29 NTM-infected individuals, 21 were females (72.4%), aged between 19–84 years (50.9±18.3 SD). There was statistically significant difference in the occurrence of *M. tuberculosis* and NTM infection between males and females (62.6% in male with TB *versus* 72.4% in female with NTM; X^2^ = 15.1; *p* = 0.0002). A total of five patients declared themselves as smokers. Roughly 72% (21/29) of patients were residents from an urban area, with 64.2% (18/21) of them having access to a water supply through piped systems. The difference between NTM urban and rural residents with access to piped water supply systems was found significant (G-test = 21.3; *p* = 0.0001).

### NTM Isolates

Eight different NTM species were identified from the 29 patients meeting the ATS criteria and included *M. massiliense* (*n* = 13; 44.8%), *M. avium* (*n* = 3; 10.3%), *M. intracellulare* (*n* = 3; 10.3%), *M. abscessus* (*n* = 2; 6.9%), *M. bolletii* (*n* = 1; 3.4%), *M. moriokaense* (*n* = 1; 3.4%), *M. fortuitum* (*n* = 1; 3.4%), *M. celatum* (*n* = 1; 3.4%) and *M. kansasii* (*n* = 1; 3.4%). Eight isolates (28%) from three patients were identified as being related to the *M. simiae* complex by 16S rRNA sequence. The sequences obtained shared 100% similarity with the corresponding 16S rRNA (GenBank accession number HM056101) and *hsp65* gene sequences (GenBank accession number HM056135) of *Mycobacterium* sp. IEC23. The pulmonary infection by *M. chelonae-M. abscessus* complex members (*M. abscessus*, *M. massiliense* and *M. bollletii*) occurred in females with an average age of 60.7 years. Among the nine patients who did not meet the diagnostic criteria the NTM disease, the NTM species isolated included *M. fortuitum* (*n* = 3; 33.3%), *M. avium* (*n* = 2; 22.2%), *M. gordonae* (*n* = 1; 11.1%), *M. colombiense* (*n* = 1; 11.1%), *M. intracellulare* (*n* = 1; 11.1%) and *M. abscessus* (*n* = 1; 11.1%).

## Discussion

About 80% (29/38) of all the NTM patients met the ATS criteria for NTM pulmonary disease [12]. Among nine cases that did not meet ATS criteria, one was highly suggestive of NTM infection. This patient showed both clinical symptoms of mycobacterial disease and a positive sputum smear. Such cases need to remain under observation and expert consultation sought [12].

This study clearly provides guidance in the diagnosis of NTM pulmonary disease in an area of high tuberculosis prevalence. Specifically, NTM-infected patients were older, more frequently female and had prior tuberculosis. In the Para state of Brazil, being of the pardo race was a risk factor for NTM disease. Roughly 70% of NTM pulmonary infections cases were patients over 50 years old, as other contemporary studies have shown [9], [19]. These data also agree with the characteristics of a series of NTM-infected patients that had revealed an increased NTM-disease susceptibility among female, slender and older individuals [20].

A variety of factors may contribute to the observation that prior tuberculosis was found to be a risk factor for NTM disease: (i) lung damage resulting from prior tuberculosis infection reduces normal clearing of pathogens; (ii) a proportion of tuberculosis patients are at increased risk for mycobacterial infection, and this subset of tuberculosis patients would be at risk for nontuberculous mycobacterial infection; and (iii) as *M. tuberculosis* infection is associated with nutritional deficiency, that subset of individuals with prior tuberculosis would be expected to be of increased susceptibility to NTM infection [21], [22], [23], [24]. COPD and cancer, diseases commonly associated with NTM disease, were less frequent in this series of case (one case of each). In the USA, COPD was described as the main co-morbidity, being found in up to 28% of the cases, while neoplastic diseases have been reported in 25% of cases [25], [26].

The fact that the average age of *M. tuberculosis*-infected patients was lower than that of the NTM patients is likely due to a number of reasons. First, *M. tuberculosis* is a highly virulent pathogen, capable of infecting healthy individuals; thus persons of all ages are susceptible. In contrast, the NTM are opportunistic pathogens; every NTM patient has some risk factor for infection. In developed countries, NTM disease is more frequently seen in older (>60 years), slender (<50 kg) men and women who lack risk factors for *M. tuberculosis* infection [20]. All risk factors for NTM disease are unknown, although it has been shown that they are innately susceptible, as they are subject to repeated NTM infection [22], [24], [27], [28], [29]. In recent publication, Dirac et al [30] reported that prior lung disease and immunosuppression appear to be associated with susceptibility to NTM disease. Furthermore, it is well-known that elderly individuals generally have a worse response to infections than the young ones, possibly as the result of the immunosenescence. This condition has been associated to an increased susceptibility to infections, including mycobacterial infections [31], [32].

A low proportion of HIV infected patients was observed among the NTM patients, as proven by serology in this study. However, this finding does not rule out the possibility of NTM-HIV co-infection in the study area, but instead it may point to the possibility of death of these patients by other causes, or perhaps even by disseminated NTM-infections, before appearance of respiratory NTM disease.

Similar results have been found in Rio de Janeiro State, where 9.8% of NTM cases were diagnosed in HIV infected patients [33]. Even smaller proportion was found in the USA and Denmark, with 3.4% and 2.4% of HIV infected patients, respectively [26], [34]. According to Sexton et al. [35], this low frequency suggests that an abnormal airway mucosa is required as initiating factor for NTM disease. Among the HIV-infected patients in this study, all of them had history of previous tuberculosis and additionally smoking, co-morbidities that predispose to NTM pulmonary disease.

NTM patients had a lower frequency of cavitary lesions compared to tuberculosis patients (Table 4). Although the radiographic features of NTM pulmonary infections are similar to those of tuberculosis, the presence of upper lobe cavitary lesions and endobronchial spread, bronchiectasis, as well as of fibroproductive nodules that change slowly, cicatricial atelectasis, and pleural thickening, were common findings in the patients of this study, which also had been shown in other studies [12], [36], [37]. The radiologic manifestations of pulmonary NTM has been classified basically as both cavitary (“classic”) or nodular-bronchiectatic (“nonclassic”) forms [37], [38], [39]. However, some NTM-cases could not be securely to fit into these categories, since they have exhibited the two forms combined. Others studies have tried to associate the patterns and forms of pulmonary lesions to NTM-species, suggesting a radiological differentiation between the diseases caused by MAC and *M. abscessus*. Briefly, has been proposed that nodular-bronquiectatic form is more frequent in patients with *M. abscessus* infection, while in those with MAC-infection the airspace consolidation and cavities are the most common findings [40], [41], [42], [43], [44], [45], [46], [47]. However, these presentations did not agree with our results in all cases.

**Table 4:** Summary of distinguishing features of NTM patients.

Characteristic	NTM	Tuberculosis	In Para population	Significance NTM vs. TB
Mean Age	52.3±17.8	38.8±17.3	27.5%	
Patients >50 years	68.9%	30.6%	14.0%	0.0001
Female	72.4%	37.4%	49.6%	<0.0001
Prior Tuberculosis	75.8%	Not Applicable	Not Applicable	
Cavitary lesions	34.5%	90.4%	Not Applicable	
Bronchiectasis	24.1%	Unknown	Not Applicable	
Pardo	89.6%	70.9%	69.5%	0.0447
Urban	72%	83.4%	68.6%	0.7988
Piped water (urban area)	64.2%	Unknown	85.3%	<0.0001

We found that the majority of the patients were of the pardo race. The percentage of the NTM-patients reported here (89.6%) is considerably higher than the percentage of pardo individuals in the state of Pará (69.5%). This could be due to either increased susceptibility of pardo individuals to NTM disease or greater opportunity of exposure to NTM-sources such as agricultural soils or drinking water [3], [48]. Assignment of increased susceptibility is problematic as pardo individuals represent a heterogeneous, genetically diverse group. In this study we found 64.2% of the patients having access to a water supply through piped systems. This information is important because, even in urban area, as in Belém – capital of the State of Pará, the water supply is still precarious, with approximately 75% of homes having access to a water supply through piped systems, being the lowest coverage of them in the peripheral urban areas, according to the 2010 Brazil Census [18]. Most patients reported in this study were residents of peripheral urban areas (data not shown).

Based in the 16S rRNA sequencing analysis, a group of five isolates was classified as *M. simiae* complex (MSC). Among the MSC members, only *M. simiae* species is recognized as potentially pathogenic to human and it is most commonly associated to cervical lymphadenitis. Nevertheless, the recovery of *M. simiae* from pulmonary specimens has been reported, especially in Israel, Cuba, and the southwestern United States [12]. Similarly, in previous study we found in our laboratory strains phylogenetically related to MSC as the most frequent NTM in pulmonary infection in Para State, Brazil [49], [50].

RGM species, including *M. abscessus*, *M. massiliense*, *M. bolletii* (formally *M. abscessus* species) represented almost 45% of all NTM pulmonary cases, whereas in Sao Paulo, *M. avium* complex (MAC) and *M. kansasii* represent the most common NTM in pulmonary disease [51], [52]. In addition, when stratifying to the NTM species level, we observed that gender associated infection was even more pronounced in the case of *M. chelonae-M. abscessus* complex (∼45% females), especially *M. massiliense* (34.5%). Griffith et al. [53] found a predominance of females (65%) among 154 cases of pulmonary disease by RGM, while descriptions of particular forms of pulmonary disease caused by MAC in women have been reported [54], [55]. Further studies are needed to elucidate the reasons for female susceptibility.

A number of factors may have contributed to the higher frequency of individuals infected with *M. massiliense* than reported in other studies. First, *M. massiliense* may be more common in the soils and waters of the Para State of Brazil. Second, *M. massiliense* is a newly described species; unknown to investigators until recently [56]. Therefore, as *M. massiliense* and *M. abscessus* share a number of common characteristics that are used for identification, earlier published studies may have misidentified *M. massiliense* isolates as *M. abscessus*. Mycobacterial taxonomy has been undergoing substantial revision; for example it has recently been shown that standard tests for identification cannot distinguish between *M. intracellulare* and the newly described *M. chimaera* [57]. That, in turn, has led to the discovery that all water isolates of *M. intracellulare* are really *M. chimaera*, forcing a re-evaluation of *M. intracellulare* epidemiology and ecology [58]. In the present instance, the recent discovery of *M. massiliense* and its separation from *M. abscessus* suggests that earlier reports around the world reporting the frequency and numbers of *M. abscessus* infections may be incorrect; the isolates could have been *M. massiliense*. Thus, prior reports that form the basis for judging that the frequency of *M. massiliense* infections in the Para State is high may be incorrect.

The results this study show that the clinical manifestations presented by the NTM-patients are suggestive of advanced disease, which reinforces the importance of the timely diagnosis of the NTM disease, since delayed treatment is associated with severe morbidity.
